# Association between worries about climate change and mental health in Norwegian adolescents

**DOI:** 10.1093/eurpub/ckac129.297

**Published:** 2022-10-25

**Authors:** M Dahhlen Granrud, T Bonsaksen, L Lien

**Affiliations:** 1 Sykehuset Innlandet HF, Norwegian National Advisory Unit on Concurrent Substance Abuse and Mental Health Disorders, Brumunddal, Norway; 2 Department of Health and Social Science, Inland Norway University of Applied Science, Elverum, Norway

## Abstract

**Background:**

Climate change has a great impact on the future of children and young people. Since the global climate strike movement, many adolescents expressed worries about climate change. But do these worries lead to declined mental health and future optimism? Thus, we aim to explore associations between prevalence of worries about climate change, leisure activities and mental health problems in a representative sample of Norwegian adolescents.

**Methods:**

In 2021, the youth survey Ungdata collected data from 139,841 Norwegian adolescents, which corresponds to a response rate of 75%. Descriptive analysis was used to calculate the prevalence of worries about climate change cannabis use and bi- and multivariate logistic regression analysis to examine the association between worries about climate change and mental health, leisure activities and alcohol and cannabis use, controlled for sociodemographics.

**Results:**

Around 37% of Norwegian adolescents are worried about climate change. Worries increased with age and are more prevalent among girls. There is a relationship between mental health problems and worries about climate change (OR = 1.80 (CI:1.75-1.86)) still after adjusting for sociodemographic variables, leisure activities and alcohol and cannabis use (OR = 1.71 (1.10-1.42)).

**Conclusions:**

The results indicate a real connection between mental health problems and worries about climate change, but the causal relationship needs further study. This knowledge makes it important for all profession, working with adolescents, paying special attention to possible negative effects of worries about climate change on the mental health of young people.

**Key messages:**

• Worries about climate change has an impact on mental health of young people.

• Adolescent’s worries about climate change should be taken seriously.

